# A Longitudinal Study of Neurocognition in Dementia with Lewy Bodies Compared to Alzheimer’s Disease

**DOI:** 10.3389/fneur.2018.00124

**Published:** 2018-03-06

**Authors:** Monica H. Breitve, Luiza J. Chwiszczuk, Kolbjørn Brønnick, Minna J. Hynninen, Bjørn H. Auestad, Dag Aarsland, Arvid Rongve

**Affiliations:** ^1^Department of Research and Innovation, Helse-Fonna Haugesund Hospital, Haugesund, Norway; ^2^Department of Geriatric Psychiatry, Clinic of Psychiatry, Helse-Fonna Haugesund Hospital, Haugesund, Norway; ^3^Faculty of Medicine, University of Bergen, Bergen, Norway; ^4^TIPS – Centre for Clinical Research in Psychosis, Stavanger University Hospital, Stavanger, Norway; ^5^Network for Medical Sciences, University of Stavanger, Stavanger, Norway; ^6^Department of Clinical Psychology, University of Bergen, Bergen, Norway; ^7^NKS Olaviken Gerontopsychiatric Hospital, Erdal, Norway; ^8^Research Department, Stavanger University Hospital, Stavanger, Norway; ^9^Department of Mathematics and Physics, University of Stavanger, Stavanger, Norway; ^10^Center for Age-Related Diseases (SESAM), Stavanger University Hospital, Stavanger, Norway; ^11^Department of Old Age Psychiatry, King’s College, Institute of Psychiatry and Neuroscience, London, United Kingdom

**Keywords:** dementia with Lewy bodies, Alzheimer’s disease, cognitive decline, longitudinal, neuropsychology

## Abstract

**Introduction:**

There are relatively few longitudinal studies on the differences in cognitive decline between Alzheimer’s disease (AD) and dementia with Lewy bodies (DLB), and the majority of existing studies have suboptimal designs.

**Aim:**

We investigated the differences in cognitive decline in AD compared to DLB over 4 years and cognitive domain predictors of progression.

**Methods:**

In a longitudinal study, 266 patients with first-time diagnosis of mild dementia were included and followed annually. The patients were tested annually with neuropsychological tests and screening instruments [MMSE (Mini-Mental Status Examination), Clinical Dementia Rating (CDR), the second edition of California Verbal Learning Test (CVLT-II), Trail Making Test A & B (TMT A & B), Stroop test, Controlled Oral Word Associations Test (COWAT) animal naming, Boston Naming Test, Visual Object and Space Perception Battery (VOSP) Cubes and Silhouettes]. Longitudinal analyses were performed with linear mixed effects (LME) models and Cox regression. Both specific neuropsychological tests and cognitive domains were analyzed.

**Results:**

This study sample comprised 119 AD and 67 DLB patients. In TMT A, the DLB patients had a faster decline over 4 years than patients with AD (*p* = 0.013). No other longitudinal differences in specific neuropsychological tests were found. Higher executive domain scores at baseline were associated with a longer time to reach severe dementia (CDR = 3) or death for the total sample (*p* = 0.032). High or low visuospatial function at baseline was not found to be associated with cognitive decline (MMSE) or progression of dementia severity (CDR) over time.

**Conclusion:**

Over 4 years, patients with DLB had a faster decline in TMT A than patients with AD, but this should be interpreted cautiously. Beyond this, there was little support for faster decline in DLB patients neuropsychologically than in AD patients.

## Introduction

Dementia with Lewy bodies (DLB) is the second most common type of neurodegenerative dementias with a prevalence of 2.2–24.7% (1). Opposed to Alzheimer’s disease (AD) which is associated with tau and amyloid pathology, DLB is an α-synucleinopathy, characterized with visual hallucinations, parkinsonism, and fluctuations. DLB seems to be a more devastating condition following a more malignant disease course than AD, with a nearly halved time until nursing home admission implying much higher costs and nearly halved survival time. Additionally, persons diagnosed with DLB experience more anxiety and sleep disturbances than persons diagnosed with AD (2–6).

Previous longitudinal studies have limited evidence base mostly due to methodological weaknesses when addressing the rate of progression of cognitive decline in DLB as compared to AD, and the data are still inconclusive. 6 of 18 studies included in our systematic review and meta-analysis reported significant differences in the rate of cognitive decline between these two conditions. Three studies reported a faster cognitive decline on Mini-Mental Status Examination (MMSE) in patients with mixed DLB and AD compared to pure forms. Two studies reported a faster decline on delayed recall and recognition in AD, and one in DLB on verbal fluency. The meta-analysis of six studies reporting MMSE scores found no significant difference in annual decline between DLB and AD (7). An updated search did not add any information, except from three studies that used MMSE as outcome. For instance, our research group found a faster decline in DLB than AD over 5 years (4.4 vs 3.2 points on average per year) (8). In the second study, patients with mixed AD and DLB were shown to decline faster than AD or DLB, and in the last one, they found some indications of a faster decline in DLB than in AD and Parkinson’s disease dementia (PDD) (9, 10).

While AD is predominantly characterized by impaired episodic memory, patients with DLB have more impaired executive, attentional, and visuospatial functioning. For example, visuospatial dysfunction is found in 71% of DLB and in 40% of AD, when controlled for motor function (11), and is found to be predictive for global cognitive impairment (12). In two studies, patients with DLB were divided into groups with high or low visuospatial functioning. Patients with low baseline visuospatial functioning had a faster decline on the Dementia Rating Scale (DRS) and activities of daily living (ADL). No differences were found in AD subgroups (12, 13). In our baseline study, we found that visuoconstructional functions in DLB were worse than in AD, but we did not find any differences between AD and DLB regarding visuoperception (14).

There is a lack of robust and reliable longitudinal studies that compare the cognitive decline in AD and DLB. Studies are needed in order to have a thorough knowledge about prognosis, a very important issue for the patients and their caregivers, as well for the community that plans caregiving. To complement previous research, we investigated (a) differences in rate of decline in neuropsychological test over 4 years in AD and DLB, (b) the association of cognitive domains at baseline and dementia progression, and (c) the associations of high and low visuospatial function at baseline and dementia progression.

## Materials and Methods

### Subjects

Totally, 266 outpatients in clinics of old age psychiatry and geriatric medicine in Western Norway with a first-time mild dementia diagnosis [MMSE score of at least 20 or Clinical Dementia Rating (CDR) 1] were recruited from 2005 and followed annually. Patients with acute delirium or confusion, terminal illness, current or previous bipolar disorder or psychotic disorder, or who were recently diagnosed with a major somatic illness were excluded (15). Follow-ups were conducted at the clinic or in nursing homes. The study protocol was approved by the Regional Committee for Medical and Health Research Ethics in Western Norway as well as in the Norwegian Social Science Data Services. Written informed consent was obtained from all participants.

### Measures

#### Dementia Diagnosis

The diagnosis of dementia was based on Diagnostic and Statistical Manual of Mental Disorders, Fourth Edition (DSM-IV) criteria. The diagnosis of Alzheimer’s disease was made according to The National Institute of Neurological and Communicative Disorders and Stroke-AD and Related Disorders Association (16). DLB was diagnosed according to the revised consensus criteria (17). Ioflupane single-photon emission computed tomography (^123^I-FP-CIT SPECT) (DaTSCAN) was conducted for 55 patients. Two independent raters set the diagnosis, and the diagnoses were evaluated after 2 and 5 years, based on all available information from follow-ups. Neuropathological confirmations are consecutively sampled. For further diagnostic information see Aarsland et al. (15).

#### Neuropsychological Measures

A battery of standardized and established rating scales and tests was used for investigating patients’ cognitive functioning. MMSE (18) is a brief test used for screening cognitive impairment. The CDR (19) is used for assessing the severity of dementia, on a global scale from 0 to 3. The second edition of California Verbal Learning Test (CVLT-II) (20) was used for assessing verbal learning and memory, in addition to MMSE delayed recall. Visual scanning, psychomotor speed, and attention were assessed with Trail Making Test A & B (TMT A & B) (21). Executive functions were also measured by MMSE attention and calculation, Stroop test (word, color, color-word, number of correct words read in 45 s) (22), and Controlled Oral Word Associations Test, using the semantic fluency task of naming animals (COWAT) (23). Naming was measured with Boston Naming Test (BNT) 15 items (24). Visuospatial function was measured by Silhouettes and Cubes on the Visual Object and Space Perception Battery (VOSP) (25) and the pentagon on MMSE. The memory domain was composed of CVLT-II List A 1–5 total, CVLT-II delayed recall and MMSE delayed recall. The executive domain of TMT A, Stroop test, COWAT naming animals and MMSE attention and calculation, and the visuospatial domain of VOSP Silhouettes and Cubes and MMSE pentagon.

Raw scores were used when analyzing neuropsychological tests. When analyzing cognitive domains, raw scores were standardized into *z*-scores, and then computed into a composite score for each domain. TMT A was reversed.

#### Statistical Analyses

Statistical analysis was made with SPSS 22.0. and R (26). Differences between AD and DLB at baseline were analyzed with Mann–Whitney *U*-test, T-test for normally distributed data, and by Pearson chi-square for categorical data. Linear mixed effects (LME) models were employed to analyze the longitudinal data to study possible differences between AD and DLB in neuropsychological tests. The LME model is to a certain extent capable of handling longitudinal data with dropout (27). Models with random intercept and random slope were used. Cox regression analyses were performed to find out if cognitive domains (memory, executive, visuospatial) at baseline were associated with survival/dementia severity. Outcome was defined as reaching CDR 3 or death. Finally, we used random effects logistic regression, finding the association between probability for CDR >1.5 (dementia severity) or lower MMSE score (cognitive decline) and as function of time and visuospatial function at baseline. High or low function was defined by a domain score above or below the mean.

## Results

### Baseline

**Figure 1:**
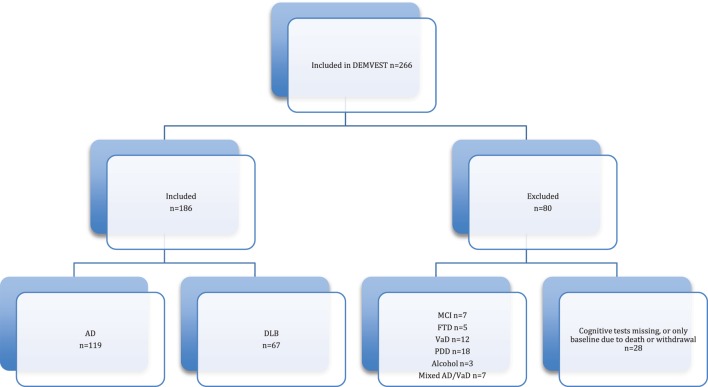
Flow chart.

Of 266 recruited patients, 186 were included in the analysis, 119 AD and 67 DLB, see Figure 1 for flow chart. There were no significant differences between the groups in age, education, depression, MMSE, and CDR scores at baseline. The AD group had significantly more women included and the DLB group had significantly longer duration of symptoms before inclusion (see Table 1).

**Table 1:** Characteristics of study group.

	AD*n* = 119	DLB*n* = 67	*p* Value^a^
Age (SD)	75.4 (7.7)	75.8 (7.3)	0.986
Sex, men/women	34/85	36/31	0.001
Education, y (SD)	9.7 (2.9)	9.6 (2.8)	0.820
MMSE (SD)	23.7 (2.4)	23.2 (3.1)	0.340
Duration of symptoms, y (SD)	1.9 (1.8)	2.5 (2.1)	0.041
Depression (SD)	1.9 (2.5)	2.4 (2.8)	0.198
CDR global, mean (SD)	0.8 (0.3)	1.0 (0.5)	0.559
Dementia medication at FU1, y/n	59/60	29/38	0.409

*^a^Differences between AD and DLB groups were analyzed using the Mann–Whitney U-test and Pearson chi-square*.

*AD, Alzheimer’s disease; DLB, dementia with Lewy bodies; MMSE, Mini-Metal Status Examination; CDR, Clinical Dementia Rating; FU1, follow up 1; y, years; y/n, yes/no*.

### Follow-up

**Figure 2:**
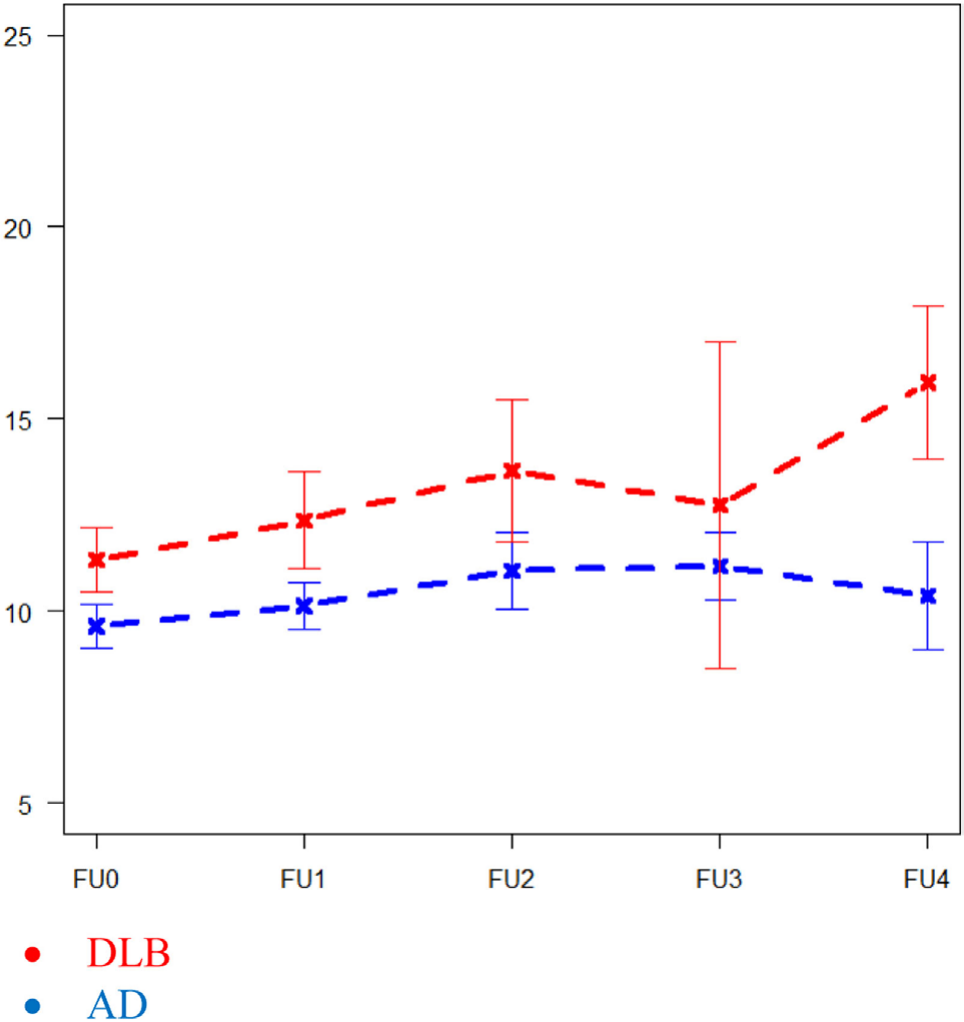
Differences in Trail Making Test A in Alzheimer’s disease and dementia with Lewy bodies over 4 years. Reported in square root transformed data.

An association between time, diagnosis, and neuropsychological tests was found on Trails A (*p* = 0.013), where the DLB group had a significantly faster decline compared to the AD group (see Figure 2). No statistical differences were found in CVLT-II, VOSP, BNT, Stroop, COWAT, and TMT A. Analyses were adjusted for sex, age, and education (see Table 2).

**Table 2:** Longitudinal changes in cognitive tests over 4 years.

	Intercept (SE)	DLB, points higher than AD at baseline (SE)	Differences in rate of decline per year (SE)	*p* Value^a^
CVLT List A 1–5 total	11.62 (2.94)	2.49 (1.33)	−0.99 (0.68)	0.149
CVLT delayed recall	−0.68 (0.83)	1.25 (0.38)	−0.04 (0.16)	0.805
VOSP silhouettes	14.15 (1.85)	−1.21 (0.85)	−0.36 (0.30)	0.239
BNT	9.72 (1.00)	−0.32 (0.46)	−0.10 (0.19)	0.595
Stroop word	55.42 (8.15)	−22.05 (3.78)	−0.21 (1.73)	0.905
Stroop Color	34.95 (6.08)	−13.94 (2.81)	−0.19 (1.24)	0.881
Stroop Color-word	10.50 (3.56)	−5.88 (1.65)	−0.76 (0.80)	0.341
COWAT animals	9.32 (1.50)	−1.45 (0.67)	−0.28 (0.30)	0.366
Trail Making Test A	150.58 (30.46)	50.08 (14.19)	−31.77 (10.98)	0.013^b^

*^a^Differences in rate between AD and DLB groups were analyzed using Linear mixed effects (LME) models*.

*^b^LME fit of square root transformed to obtain a satisfactory fit*.

*AD, Alzheimer’s disease; BNT, Boston Naming Test; CVLT-II, California Verbal Learning Test, second edition; COWAT, Controlled Oral Word Associations Test; DLB, dementia with Lewy bodies; VOSP, Visual Object and Space Perception Battery*.

Higher executive domain scores at baseline were associated with a longer time to reach severe dementia (CDR 3) or death for the total sample (*p* = 0.032), but type of dementia were not. No such associations were observed for the visuospatial or memory domain. The analyses were adjusted for age and sex (see Table 3). We also analyzed if high or low visuospatial function at baseline in AD and DLB were associated with cognitive decline (MMSE) (*p* = 0.108) or dementia severity CDR (*p* = 0.654), but no differences were found. The analyses were adjusted for sex, age, and education.

**Table 3:** Associations between cognitive domains and negative outcome.

	Associations	Group-dependant associations
Executive	0.032	0.321
Memory	0.236	0.602
Visuospatial	0.152	0.621

*^a^Associations were analyzed with Cox regression and p-values are given*.

*AD, Alzheimer’s disease; DLB, dementia with Lewy bodies*.

At one-year follow-up, only 11.9% (*n* = 8) of the DLB group were able to complete TMT B, compared to 27.7% (*n* = 33) in the AD group, therefore, not included in the analyses.

## Discussion

Patients with DLB had a faster decline on TMT A compared to patients with AD over four years. Higher executive function at baseline was associated with a slower progression of dementia and longer survival in the total group, but no differences between AD and DLB were observed. We did not find any association between high or low visuospatial function at baseline and the rate of decline.

The TMT A task is relatively undemanding cognitively and motor tempo determines the performance to a degree. The faster decline in the performance of the DLB group could be due to more deficiencies in motor and visuospatial functions, which are more prominent in DLB than in AD. On the other hand, the two groups were progressing without any significant difference on other neuropsychological test, some of which were also depending on such skills. Previous studies have not found differences in decline over time between the two groups in TMT A, and due to missing data and the risk of familywise statistical error, our findings should be cautiously interpreted. In our baseline study, we found that DLB patients performed worse than AD on tests correlating with parkinsonism, not with visual hallucinations or cognitive fluctuation (14).

High executive functions at baseline were associated with a slower progression of reaching severe dementia or death for the total sample, but there were no differences in progression between AD and DLB. In the memory or visuospatial domains, we found no significant differences. Chunking single neuropsychological tests that are assumed to measure more or less the same cognitive factor into one cognitive domain has pros and cons. We can rely on more tests and presumably a more robust measure, nevertheless the domains, especially the executive, are not entities, but can be broken into several functions (28). This could be the reason why studies of mild cognitive impairment have shown mixed results regarding the link between executive dysfunctions and prediction of conversion to dementia (29). On the other hand, we have not found any studies that have investigated the difference in cognitive domains over time in AD and DLB. As executive functions also encompass several forms of attention, included fluctuations, one of the core symptoms of DLB, it would be interesting to find out if patients with DLB and relatively preserved executive functions have milder fluctuations and a slower disease progression.

Previous research has shown that patients with DLB and low visuospatial function have a faster decline on cognitive screening tests or ADL function (13). We could not confirm their findings in our study. This could possibly be due to longer follow-up time, the tests that are used in the analyses, or the lack of neuropathological reports on the total sample. Without the neuropathological confirmation of the diagnosis, we cannot rule out the possibility that there are more DLB cases with neocortical or nigral predominant type of LB, and not the limbic type that is more often associated with more rapid decline in visuospatial function (30). At the same time in AD, the zeitgeist is to move away from using the concept AD, and instead refer to the pathology behind the symptoms, since there are several subtypes and other AD mimicking diseases (31). Lack of systematical differences could hypothetically be due to grouping AD patients with mixed pathologies that are not identical and the expression of the same disease. We also lack information about genetics that could have affected the cognitive decline over time.

In clinical practice, the two most frequent and important questions for people newly diagnosed with dementia are “is there anything that can prevent progression?” followed by “how long will it take before I lose my mind?” This is not easy to predict for the individual patient, but on group levels, for patients with relatively preserved executive functions, the progression seems to be slower. Even though there are few studies on neuropsychological tests that predict the rate of decline in AD (32), our finding is supported from studies that found impaired executive functions to be associated with a faster decline (33, 34).

Thus, there is no persuasive evidence that there is a dissimilar decline in neuropsychological tests and cognitive domains between AD and DLB. On the other hand, recent studies of good quality have found that DLB progress faster than AD and PDD using the cognitive screening test MMSE, as outcome. Our clinical impression of a more rapid decline in patients with DLB is evident in previously mentioned research, probably caused by the α-synuclein pathology and the severe BPSD symptoms that are highly frequent in DLB. The cognitive differences though, is not the dominant predictor of a more rapid disease course (8).

There are some limitations in the study. We have previously argued for longer follow up time than 1 year in these kinds of studies (7). Regardless a large sample size at baseline, missing data is a common issue in longitudinal studies with neurodegenerative diseases due to cognitive decline and death. In our study, LME models were used to minimize this problem. Also, we used neuropsychological test scores at baseline with non-cognitive measures as outcome. Another advantage of this approach is that in the beginning of the dementia, AD and DLB are more separable, since the clinic and brain pathology is more specific, not widespread and mixed with brain pathology that comes as a natural consequence of aging.

Mini-Mental Status Examination was the only test that all the patients could complete at every follow up. MMSE has been criticized for not being sensitive enough for measuring changes in patients with pure DLB since they have a different cognitive profile than AD patients, but Lessig et al. found it valid for Parkinson’s disease patients who share most of the same pathology (35).

The mean score at baseline in CVLT-II delayed memory was low (mean 1.3 for AD and 2.5 for DLB out of a maximum score of 16) and missing statistically significant differences could be due to floor effect. There is also a potential problem with circularity, since the diagnoses are made partially by relying on results on cognitive tests (i.e., memory impairments for AD). However, the autopsy results indicate that the diagnoses are not biased by circularity (36).

Strengths of the study are the longitudinal design with one of the largest samples of DLB patients which has been followed annually, and the AD and DLB groups were similar in age, education, and MMSE score at baseline. The patients are thoroughly evaluated and diagnosed, and the majority of the DLB patients have undergone ^123^I-FP-CIT SPECT (DaTSCAN), and DLB is, therefore, not likely to be underdiagnosed in the study group. Autopsy has been done in 43 cases with 92% sensitivity and 83% specificity for clinical diagnosis (36).

## Conclusion

Over 4 years, there was little support for the hypothesis that patients with DLB had faster decline neuropsychologically than patients with AD. The only exception was TMT A, where patients with DLB had a faster decline than AD patients, but this should be interpreted cautiously due to missing data and the risk of familywise statistical error. Higher executive domain scores at baseline were associated with a longer time to reach severe dementia or death for the total sample, but type of dementia was not. Further research is needed and information about genetics should be included.

## Ethics Statement

This study was approved by the Regional Committee for Medical and Health Research Ethics in Western Norway and the Norwegian Social Science Data Services. All subjects gave written informed consent in accordance with the Declaration of Helsinki.

## Author Contributions

MB and LC participated in the data acquisition, made the hypothesis, analyzed and interpreted data, and drafted the manuscript. KB made the hypothesis, interpreted data, and revised the manuscript. BA performed and interpreted the longitudinal analysis, and revised the manuscript. MH contributed with the hypothesis, interpretation of the analysis, and revised the manuscript. DA and AR participated in the data acquisition, contributed with the hypothesis, interpretation of the analysis, and revised the manuscript. All authors read and approved the final version of the manuscript.

## Conflict of Interest Statement

The authors declare that the research was conducted in the absence of any commercial or financial relationships that could be construed as a potential conflict of interest.

## Abbreviations

AD, Alzheimer’s disease; ADL, activities of daily living; BNT, Boston Naming Test; BPSD, behavioural and psychological symptoms; CDR, Clinical Dementia Rating; CVLT-II, the second edition of California Verbal Learning Test; COWAT, Controlled Oral Word Associations Test; DLB, dementia with Lewy bodies; DRS, Dementia Rating Scale; DSM-IV, Diagnostic and Statistical Manual of Mental Disorders, Fourth Edition; LB, Lewy bodies; LME, linear mixed effects; MCI, mild cognitive impairment; MMSE, Mini-Mental Status Examination; PD, Parkinson’s disease; PDD, Parkinson’s disease dementia; TMT A & B, Trail Making Test A & B; VOSP, Visual Object and Space Perception Battery; ^123^I-FP-CIT SPECT, ioflupane single-photon emission computed tomography.
